# Supplementary material for: Synthetic ZFTA fusions pinpoint disordered protein domain acquisition as a mechanism of brain tumorigenesis
Source: Nat Cell Biol. 2025 Aug 27;27(9):1496–509. doi: 10.1038/s41556-025-01745-3 (PMC12431856; doi:10.1038/s41556-025-01745-3)
Supplement: Supplementary file 7 — Unprocessed images of gels and blots. [file 41556_2025_1745_MOESM7_ESM.pdf]

A

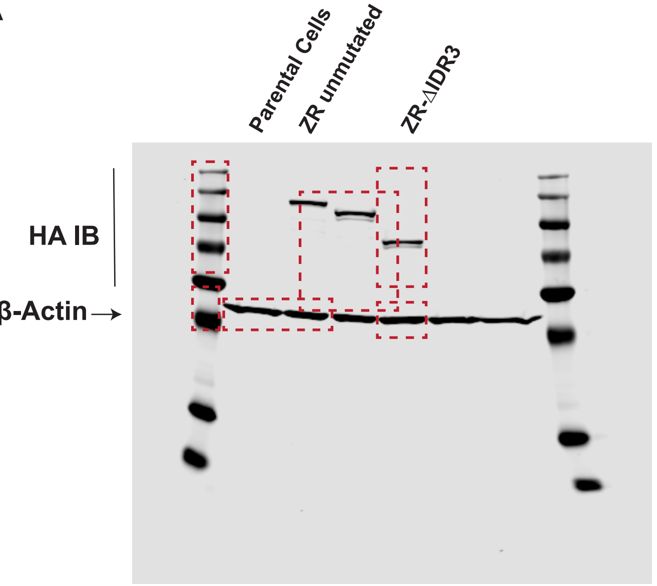

(Used in Extended Data Fig.5D, E)
(Areas indicated by red are used in the figure)

B

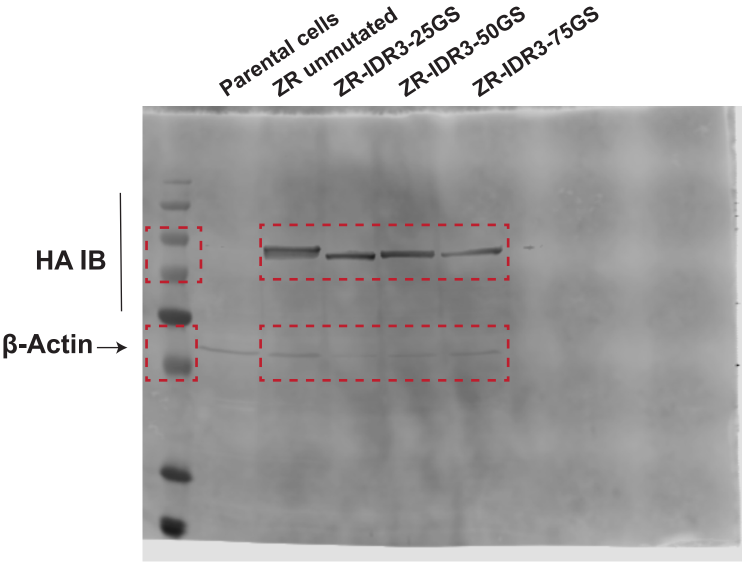

(Used in Extended Data Fig.6C)
(Areas indicated by red are used in the figure)

C

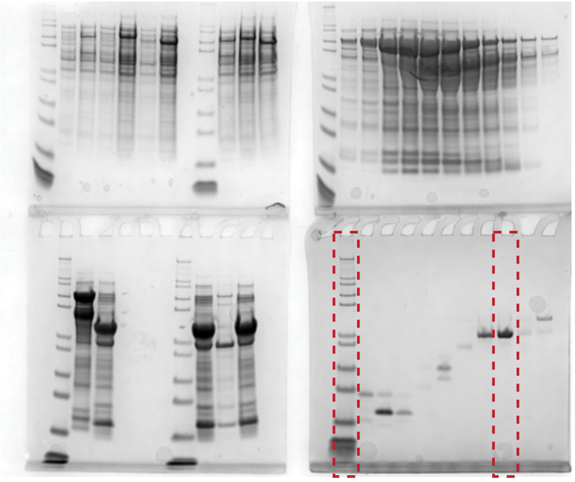

(Used in Fig.3I, top panel, coomassie stained gel)
(Areas indicated by red are used in the figure)

D

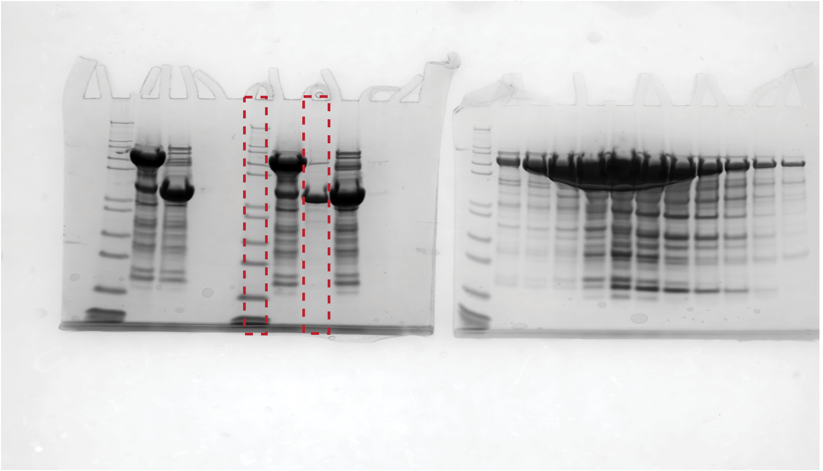

(Used in Fig.3I, bottom panel, coomassie stained gel)
(Areas indicated by red are used in the figure)

E

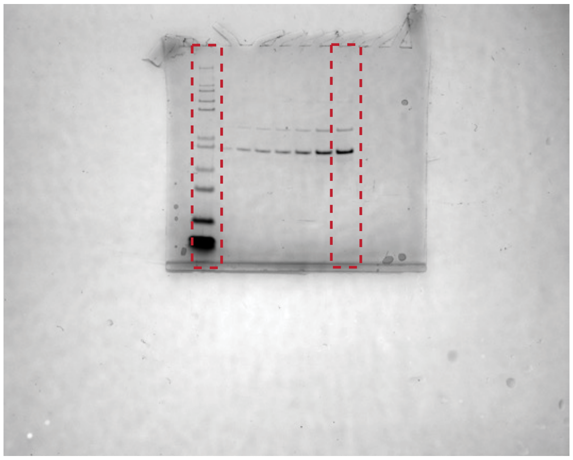

(Used in Extended Data Fig.4G, coomassie stained gel)
(Areas indicated by red are used in the figure)
